# Inactivation of Zika Virus by Photoactive Iodonaphthyl Azide Preserves Immunogenic Potential of the Virus

**DOI:** 10.3390/pathogens8040188

**Published:** 2019-10-12

**Authors:** Amy L. Austin, Bianca Galasso, Caitlin Nickens, Barbara Knollmann-Ritschel, Anuj Sharma

**Affiliations:** Department of Pathology, Uniformed Services University of the Health Sciences, Bethesda, MD 20853, USA; amy.l.austin20.mil@mail.mil (A.L.A.); bianca.galasso.ctr@usuhs.edu (B.G.); caitlin.m.nickens.mil@mail.mil (C.N.); barbara.knollmann-ritschel@usuhs.edu (B.K.-R.)

**Keywords:** Zika virus, vaccine, inactivation, iodonaphthyl azide, immunization

## Abstract

Zika virus’s (ZIKV) emergence as a pathogen of significant public health importance has accelerated efforts to develop a ZIKV vaccine. To date, the need for an effective ZIKV vaccine is unmet. In this study, we report inactivation of ZIKV using a hydrophobic photoactive compound: 1, 5 iodonaphthyl azide (INA). 50 and 100 µM of INA completely inactivated ZIKV (INA-ZIKV). Western blot and ELISA analysis show some loss of the binding capacity of INA-iZIKV to anti-ZIKV monoclonal antibodies; however, immunization of mice with INA-iZIKV demonstrated seroconversion and ZIKV-neutralizing antibody response. RNA isolated from INA-iZIKV did not induce productive infection in Vero cells, suggesting inactivation of ZIKV RNA. These results suggest that in the absence of an approved ZIKV vaccine, INA-iZIKV can be pursued as a viable ZIKV vaccine candidate.

## 1. Introduction

ZIKV was first identified in the Zika forest in Uganda in 1947. Exposure to ZIKV was known to occur in Africa and Asia; however, the virus was not associated with clinically relevant disease in humans until 2007. Beginning in 2007–2008, ZIKV emerged as a pathogen of significant public health importance as it caused widespread outbreaks of disease throughout the tropics [1]. The major impact of ZIKV infection occurs during pregnancy, when the virus can cross the placental barrier and cause severe adverse effects in the developing fetus such as microcephaly, calcification, developmental disorders, and stillbirth [2]. The Zika Outcomes and Development in Infants and Children study on children born with microcephaly or evidence of ZIKV infection found a range of disabilities and developmental disorders including motor, vision, and hearing abnormalities, as well as seizure disorders and functional limitations requiring lifelong assisted care [3]. Although the number of new cases of autochthonous infection of ZIKV started to decline in 2018, populations which have not yet experienced a large local outbreak of the disease, such as in the U.S., are still susceptible to explosive outbreaks [4]. New outbreaks of ZIKV in tropical countries suggests persistent circulation of the virus, which, combined with the endemic transmission vector, *Aedes* spp., present a significant risk of larger ZIKV outbreaks [5,6,7]. Therefore, effective prophylactic measures are needed to counter these potential outbreaks of ZIKV. Several candidates of ZIKV vaccine are in development, but to date none have received FDA approval or licensure [8,9,10,11,12]. In this study, we describe inactivation of ZIKV using the photoactive hydrophobic compound INA. INA, by its hydrophobic property, sequesters in the hydrophobic domain of the lipid bilayer and, upon photoactivation by long-wavelength UV rays, covalently binds to the transmembrane domain of the proteins, resulting in their functional inactivation [13,14,15]. This approach has been used to inactivate human immunodeficiency virus, Venezuelan equine encephalitis virus, dengue virus, and chikungunya virus [16,17,18,19]. 

## 2. Results

### 2.1. ZIKV Is Inactivated by INA

**Figure 1:**
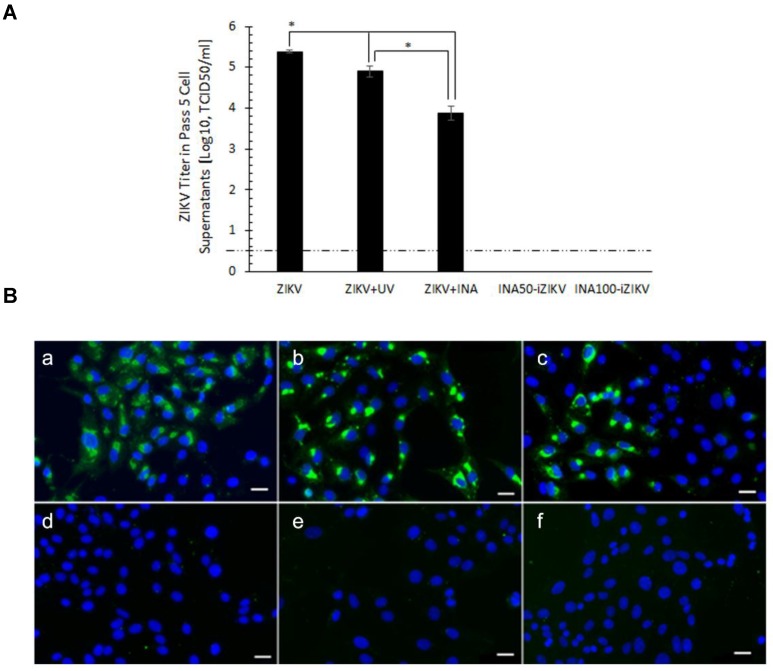
INA inactivated ZIKV: (**A**) Virus titer in the supernatants of Vero cells infected at fifth serial passage of the inactivated and control ZIKV. The virus was detected in the supernatant of cells infected with live ZIKV or ZIKV exposed to UV only (ZIKV+UV), or ZIKV treated with 100 µM INA (ZIKV + INA). The virus titers in supernatants of cells infected with ZIKV+UV and ZIKV+INA were significantly lower than the live ZIKV (* *p* < 0.05) group. No virus was detected in the supernatants of cells infected with the ZIKV inactivated with 50 or 100 µM of INA in the presence of UV (INA_50_-iZIKV and INA_100_-iZIKV, respectively). Values are expressed as Mean ± SD. (**B**) Vero cells infected with the control and test samples were evaluated for ZIKV infection by immunofluorescence assay using anti-ZIKV monoclonal antibody, ZV-54. (**a**) Live ZIKV; (**b**) ZIKV+UV; (**c**) ZIKV+INA; (**d**) INA_50_-iZIKV; (**e**) INA_100_-iZIKV; (**f**) uninfected Vero cells. The bar in figure insert B corresponds to 50 µm.

Sucrose gradient-purified ZIKV was inactivated by INA as described before [18,20]. Inactivation of ZIKV by INA was verified by five serial passages of the inactivated virus in Vero cells. Serial passaging of viruses in a host cell line is a sensitive method of identifying virus particles that may have escaped inactivation [17,21]. Ten percent of the inactivated volume was used for the 1^st^ passage, thereafter 50% of the cell supernatant was transferred for each subsequent passage. The following control and test samples were passaged: Live ZIKV (ZIKV); ZIKV exposed to the UV rays without addition of INA (ZIKV + UV); ZIKV exposed to INA at a final concentration of 100 µM without additional exposure to UV rays (ZIKV + INA); ZIKV treated with 50 or 100 µM INA and exposed to UV rays (INA_50_-iZIKV and INA_100_-iZIKV, respectively). No cytopathic effect was observed in cells infected with INA-iZIKV (50 and 100 µM INA) in the fifth passage. As expected, the positive infection control demonstrated cell rounding and detachment, and loss of cell monolayer (Supplementary Figure S1). Virus titer in the supernatants confirmed lack of virus replication in INA-iZIKV samples (Figure 1A). ZIKV-specific staining was performed on Vero cells infected with the inactivated and control-treated virus. No virus-specific immunofluorescence was observed in cells infected with INA_50_-iZIKV or INA_100_-iZIKV (Figure 1B). Therefore, complete inactivation of ZIKV by INA is suggested by lack of escape/residual virus infectivity in serial passages and non-replication of ZIKV in sensitive Vero cells. The inactivation was dependent on the binding of INA to ZIKV induced by exposure to UV rays. From here onwards, only INA_100_-iZIKV was used in the experiments as INA-inactivated ZIKV (INA-iZIKV). 

### 2.2. RNA Isolated from INA-iZIKV Is Non-infectious

**Figure 2:**
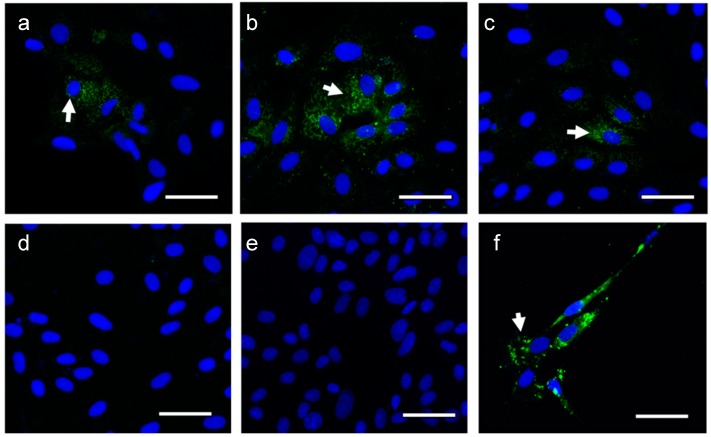
Vero cells transfected with RNA isolated from INA-inactivated ZIKV: Vero cells were transfected with RNA isolated from various test and control samples. (**a**) ZIKV-RNA; (**b**) ZIKV+100 µM INA-RNA; (**c**) ZIKV+UV-RNA; and (**d**) INA_100_-iZIKV (ZIKV+100 µM INA+UV)-RNA. (**e**) Uninfected cells and (**f**) live-ZIKV infection served as negative and positive controls of infection, respectively. Arrows indicate ZIKV specific staining. The bar corresponds to 50 µm.

The positive sense single-stranded RNA genome of flaviviruses is infectious and, when transfected into cells, can initiate virus infection. Earlier, we have shown that the similar positive sense single-stranded RNA genome of the alphavirus is inactivated by INA inactivation of the virus [17]. To test if INA_100_-iZIKV RNA is similarly inactivated by INA inactivation of the virus, Vero cells were transfected with RNA isolated from the inactivated virus and controls. Immunofluorescent staining for ZIKV antigen in Vero cells transfected with RNA isolated from INA_100_-iZIKV did not show virus specific E-protein staining (Figure 2d), which was observed in Vero cells transfected with ZIKV-RNA (Figure 2a). Vero cells transfected with RNA isolated from ZIKV treated either with UV or INA alone were also positive for ZIKV specific immunofluorescence (Figure 2b,d). Uninfected cells and Vero cells infected with live ZIKV served as negative and positive control for infection, respectively (Figure 2e,f). The data suggests inactivation of ZIKV RNA-induced infectivity. 

### 2.3. INA-iZIKV Binds to Anti-ZIKV Monoclonal Antibodies 

**Figure 3:**
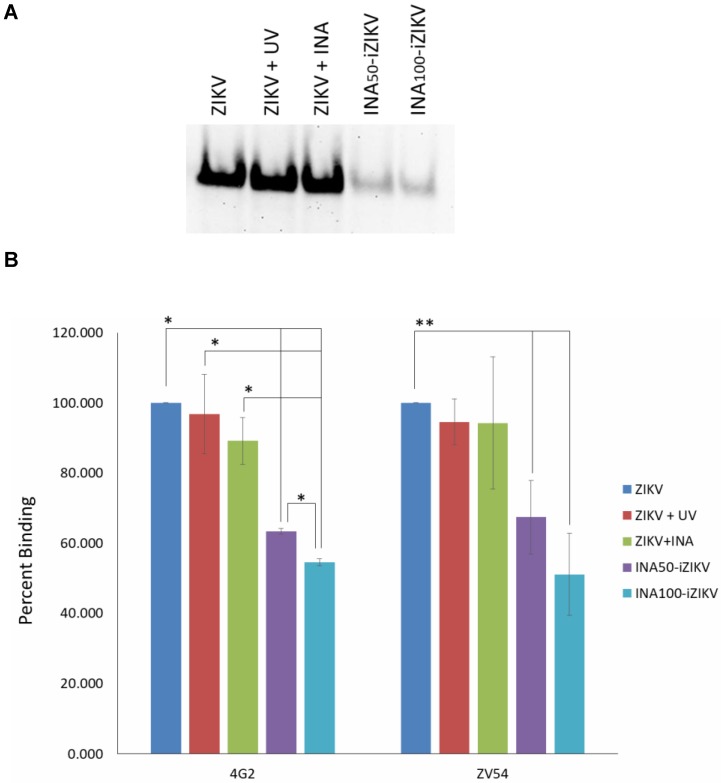
Binding of INA-iZIKV to anti-ZIKV antibody: (**A**) Binding of the test and control ZIKV samples to ZIKV-neutralizing antibody, ZV-54 (anti-ZIKV E-protein) was tested under reducing condition by Western blot analysis. Binding of INA_50_-iZIKV and INA_100_-iZIKV was found to reduce compared to the live ZIKV or ZIKV treated with INA or exposed to UV rays alone. (**B**) Binding of INA-iZIKV to two ZIKV-neutralizing antibodies, ZV-54 and 4G2, under native form was determined by ELISA. Similar to western blot data, binding of INA_50_-iZIKV and INA_100_-iZIKV to neutralizing antibodies was significantly reduced compared to live ZIKV control. Percent binding is expressed relative to binding of live-ZIKV to the respective antibodies. Values are presented as Mean ± SD. * *p* value < 0.01; ** *p* value < 0.05.

Binding of inactivated virus to neutralizing antibodies can indicate the preservation of neutralizing epitopes on an inactivated virus. Therefore, binding of INA-iZIKV with monoclonal neutralizing antibodies was tested by Western blot and ELISA. Western blot analysis of INA-iZIKV using the ZIKV specific neutralizing antibody ZV-54 showed binding of INA_50_-iZIKV and INA_100_-iZIKV to ZV-54 albeit at reduced levels compared to non-inactivated ZIKV (Figure 3A). The reduction in binding capacity of ZIKV to ZV-54 was specific to binding of INA to ZIKV proteins as non-UV INA-treated ZIKV and UV exposed ZIKV did not show reduction in binding of the virus to ZV-54. Further evaluation of binding of native form of INA-iZIKV to antibodies was evaluated using ELISA. Similar to Western blot data, binding of INA-iZIKV to ZIKV neutralizing antibodies (4G2 and ZV-54) was observed (Figure 3B). As seen in Western blot, significant reduction in binding of INA-iZIKV to neutralizing antibodies was observed, which was dependent on the covalent binding of INA to ZIKV (Figure 3B). No significant difference in the binding capacity of ZV-54 and 4G2 to ZIKV treated with UV alone or INA alone was observed. In conclusion, INA-iZIKV was recognized by the neutralizing antibodies, however, binding capacity of the neutralizing antibodies to the inactivated virus was reduced. 

### 2.4. Immunization with INA-iZIKV Induces Anti-ZIKV Antibody Response

**Figure 4:**
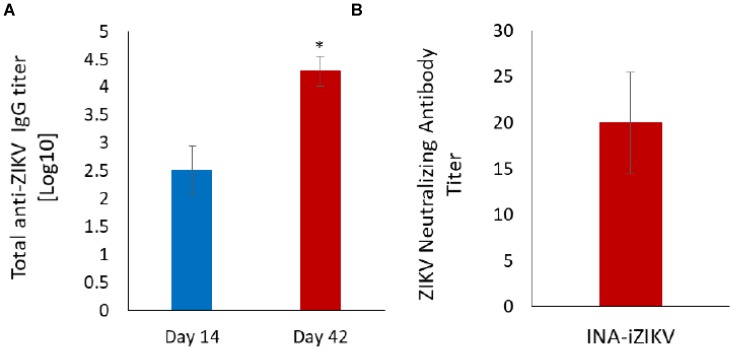
Anti-ZIKV antibody response in mice: (**A**) Total anti-ZIKV IgG titer were determined by ELISA. All five animals seroconverted on day 14 post immunization and total anti-ZIKV IgG titer increased significantly two weeks after booster immunization. (**B**) ZIKV-neutralizing antibody titer as determined by standard PRNT assay in serum samples collected two weeks after booster immunization. Values are presented as Mean ± SD.

To determine the induction of protective immune response by INA-iZIKV, C57BL6 mice were immunized with 10 µg of the inactivated INA_100_-iZIKV on day 0 and again on day 28. As there is no significant difference between the inactivation efficacy and binding of ZIKV inactivated by 50 or 100µM INA, INA_100_-iZIKV was used in these experiments. Total anti-ZIKV IgG was evaluated on day 14 and day 42 by ELISA. All mice seroconverted following the first immunization dose and the total anti-ZIKV IgG titer increased significantly two weeks after the booster dose (Figure 4A). No binding of pre-immune serum samples with ZIKV was observed at the lowest dilution of 1:20. Neutralizing antibody titer measured as PRNT50 showed 1:20 titer two weeks after booster dose (Figure 4B). No neutralization of ZIKV virus was observed with pre-immune serum at 1:10 serum dilution. 

## 3. Discussion

INA has been shown to inactivate enveloped and non-enveloped viruses, including flaviviruses [17,18,19,20,22]. ZIKV was completely inactivated by INA, including the loss of the infectivity of viral RNA. The serial passage of the inactivated virus *in-vitro* is a sensitive method to identify residual escape virus particles and determine inactivation of the virus formulation. A study by Shuxuan et al., 2018 showed that in 1 day old C56BL/6 mice, the LD_50_ (intra peritoneal) of ZIKV was 3.72 × 10^1^ TCID_50_ [23]. In our study, there was no detectable virus after five serial passages of the INA-inactivated ZIKV, therefore, it is highly likely that a complete inactivation of ZIKV was achieved by INA. However, serial passaging in sensitive neonatal mice models is considered the gold standard for determining the inactivation of the virus [17]. In future studies, serial passage of INA-inactivated ZIKV will need to be performed in neonatal mice to confirm complete inactivation. Reduction in the binding of INA-inactivated viruses with their respective antibodies has been described previously [17,18,19,20,22]. INA binds to the cysteine amino acid residues in proteins [24]. Although the lateral-ridge region of domain III (DIII) of ZIKV E-protein that binds with the ZV-54 antibody does not have a cysteine residue, there are two cysteine residues in this sequence that are present adjacent to the amino acid sequence that interacts with the antibody ZV-54 [25]. It is possible that binding of INA to these cysteine residues may have interfered with the binding of monoclonal antibodies to their respective region on ZIKV E-protein [22]. Despite reduction in the binding of INA-iZIKV to monoclonal neutralizing antibodies, in this proof-of-concept study, we demonstrate immunization with INA-iZIKV induced anti-ZIKV IgG and ZIKV-neutralizing antibodies. Our study is limited by the lack of protective efficacy against live-ZIKV challenge; however, the total anti-ZIKV IgG and ZIKV-neutralizing antibody titers observed in our study are comparable to the demonstrated protective threshold levels of anti-ZIKV IgG and ZIKV-neutralizing antibody titers [26]. Although variations in methods between different laboratories make it difficult to directly compare the absolute protective titers of neutralizing antibodies, the data nevertheless shows that INA-inactivated ZIKV is immunogenic *in-vivo*. Future studies including comparison to the other forms of virus inactivation and dose-sparing studies, which were beyond the scope of this study, will determine the optimum dose of INA-iZIKV immunization that can induce further elevated levels of neutralizing antibodies and demonstrate protective efficacy of INA-iZIKV against live ZIKV challenge. The two independent mechanisms of action of INA, i.e., the inactivation of viral envelop proteins and inactivation of the positive-sense RNA genome of the virus, will provide a better safety profile to the INA-inactivated ZIKV vaccine. Our proof of concept study, therefore, provides strong basis for further development of INA-iZIKV as vaccine. 

## 4. Methods

### 4.1. Virus and Inactivation

ZIKV, strain PRVABC59, 2015 Puerto Rico isolate (BEI Resources, Manassas, NA, USA; GenBank: KX087101), was grown in Vero cells (passage 5) and purified on a 15–50% sucrose gradient. Inactivation of ZIKV was performed as described before [17] with slight modification of the omission of glutathione from protocol because ZIKV was found to be inactivated by 20 mM glutathione alone (data not shown). This observation is similar to the inactivation of encephalomyocarditis virus by glutathione observed in our previous study [22]. Virus stock was suspended in 1X DPBS at a protein concentration of 0.38 mg/mL in a clear transparent tube (Sarstedt, Nümbrecht, Germany). From this point, reduced light conditions were used. INA (10 mM stock in DMSO) (Biotium, Fremont, CA, USA) was added to the virus suspension for a final concentration of 50 and 100 µM in 2–3 installments to avoid precipitation. The samples were incubated for 20 minutes in the dark at room temperature. Samples were then centrifuged at low speed (1000 rpm) for one minute to remove any INA crystals that precipitated in the aqueous solution. The supernatant was then transferred to a new tube. The virus suspension was irradiated using a 100 W mercury UV lamp (UVP, Upland, CA, USA) in the following setup: A clear glass plate, which acted as short wavelength UV ray filter, was placed immediately in front of the lamp and a double-distilled water filled transparent T-75 tissue culture flask (Corning, Corning, NY, USA) was placed 6 cm away from the glass plate to act as a heat filter. Samples were placed 6 cm away from the flask in such a way that samples were completely illuminated with the light passing through the flask. Irradiation was done for 120 seconds, vortexed, irradiated again for 120 seconds, vortexed, and then irradiated a final time for 60 seconds. After this point, full light conditions were used. Samples were stored at −20°C until further use. 

### 4.2. Serial Passaging of Inactivated Virus In-Vitro 

Vero cells were plated in 12-well plates (Corning, Corning, NY, USA) at a cell density of 50,000 cells/well and cultured overnight. 30 µL of the samples ZIKV + INA, ZIKV + UV, INA_50_-iZIKV, INA_100_-iZIKV, and ZIKV, which is equivalent to 10% of the inactivated virus (0.8 multiplicity of infection (MOI)) were added to the wells in duplicate and incubated for 5 days, at which point the supernatants were collected and 500 µL of each supernatant was passaged to a corresponding well in a new 12-well plate of Vero cells (1:1 dilution in cell culture medium). The supernatants were passaged 5 times total. After the serial passaging was complete, the infectious titer of the supernatants was evaluated as 50% tissue culture infectivity dose (TCID 50/mL) using the Reed and Muench method as described before [27]. 

### 4.3. Immunofluorescence

Vero cells grown in 8-chamber slides (EMD Millipore, Burlington, MA) were infected with 10% volume of the test and inactivated ZIKV samples and incubated for 3 days post infection. Cells were fixed with ice cold acetone:methanol (1:1 v/v) for 10 min followed by 3 rinses with 1XPBS. Blocking was performed with 1% Bovine Serum Albumin (Sigma Aldrich, St. Louis, MO, USA) for 1 h at room temperature. Slides were then incubated with anti-ZIKV antibody ZV-54 (EMD Millipore, Burlington, MA, USA) diluted at 1:200 in blocking buffer for 1 h at 37 ℃. Slides were washed 3 times, 5 min per wash, with 1X PBS and incubated with AlexaFluor 488-conjugated anti-mouse IgG (Invitrogen, Carlsbad, CA, USA) diluted at 1:500 in blocking buffer for 1 h at 37 ℃. Slides were washed with 1X PBS as mentioned above and mounted using Vectashield mounting medium containing DAPI (Vector Labs, Burlingame, CA, USA).

### 4.4. Western Blot Analysis of INA-iZIKV

Binding of INA-iZIKV under reducing conditions was determined by Western blot analysis as previously described [18]. Samples (3.8 µg/sample) were run on a NuPAGE 4–12% Bis-Tris gel (ThermoFisher Scientific, Waltham, MA, USA) under reducing conditions and transferred to a nitrocellulose membrane (Bio-Rad, Hercules, CA, USA). Membrane was blocked using 3% non-fat dry milk (Santa Cruz Biotechnology, Dallas, TX, USA) in TBS-T (1X TBS (Fisher Scientific, Waltham, MA, USA) + 0.5% Tween 20) for 1 h at room temperature. The primary antibody, ZV-54, was added (8 µL/10 mL of blocking buffer) and incubated overnight at 4 °C. The membrane was then washed 3 times, 5 min per wash, with TBS-T. HRP-conjugated goat anti-mouse IgG (Cell Signaling Technology, Danvers, MA) (2 µL/10 mL of blocking buffer) was added onto the membrane and incubated for 1 h at room temperature followed by 3 washes with TBS-T. Chemiluminescence was developed using 8 mL of ECL substrate (GE Healthcare, Chicago, IL, USA). 

### 4.5. ELISA to Determine Binding of Anti-ZIKV Antibodies to Native form of INA-iZIKV

A 96-well ELISA plate (Santa Cruz Biotechnology Cat# sc-204463) was coated with 100 ng/50 µL/well of each of the test and control virus samples suspended in 1X PBS for overnight at 4 °C. Wells were then washed 6 times with approximately 300 µL of wash buffer (1X PBS-T: PBS [Quality Biological, Gaithersburg, MD, USA] with 0.5% Tween 20) followed by blocking with 100 µL/well of 3% non-fat dry milk [Santa Cruz Biotechnology, Dallas, TX, USA] in PBS-T for 1 h at room temperature. Blocking buffer was removed and 100 µL/well of 1:200 diluted ZV-54 antibody or D1-4G2-4-15 (4G2) pan-flavivirus antibody (BEI Resources, Manassas, VA, USA) in blocking buffer was added. 100 µL/well of blocking buffer alone was added as the negative control. The plate was incubated for one hour at 37 °C. Wells were washed 6 times with PBS-T. 100 µL of 1:500 diluted goat anti-mouse HRP conjugated antibody [Cell Signaling Technology, Danvers, MA] in blocking buffer was added to each well and the plate was incubated for 1 hour at 37 °C. Plate was washed 6 times with PBS-T. The plate was then incubated with 100 µL of HRP substrate [SeraCare Life Sciences Inc., Milford, MA, USA] for 30 minutes at room temperature until blue color developed. 100 µL of stop solution was then added to each well. Absorbance was read at 600 nm. 

### 4.6. RNA Isolation and Transfection 

RNA was isolated from various experimental groups using the MagMax Viral/Pathogen Nucleic Acid Isolation Kit (ThermoFisher Scientific, Waltham, MA, USA) as per the manufacturer’s protocol. Briefly, 50 µL of sample was diluted to 200 µL with sterile PBS and mixed with activated nucleic acid binding magnetic beads and proteinase K. Samples were mixed at 300 rpm for 5 min followed by incubation at 65 °C for 10 min and an additional 5 min mixing at 300 rpm. Beads were collected on the side of tube using a magnetic stand for 5 min. Supernatant was carefully removed and beads were washed with wash buffer included in the kit followed by two washes with 80% ethanol. Final elution was done in 60 µL of nuclease free water. RNA was quantified using NanoDrop (ThermoFisher Scientific, Waltham, MA, USA) and stored at −20 °C until further use. Transfection of Vero cells was performed in 8-chamber slide (EMD Millipore, Burlington, MA, USA), with equal volumes of RNA for each sample using siPORT NeoFx transfection reagent (ThermoFisher Scientific, Waltham, MA, USA). Cells were fixed 4 days post-transfection and virus infection was visualized by immunofluorescence staining as described above. 

### 4.7. Immunization of Mice with INA-iZIKV 

Five week old C57Bl6/J female mice (Jackson Laboratories) were immunized with 10 µg of INA-iZIKV (antigen concentration back-calculated using stock virus concentration) by intramuscular (i.m.) injection using 0.5 cc insulin syringes fitted with 31G 8 mm needles (BD Biosciences, San Jose, CA, USA). Primary and booster immunizations were administered on day 0 and day 28, respectively. Control mice were similarly injected with sterile saline. Mice were bled on day 14 by tail vein nick and day 42 by cardiac puncture and serum was isolated using the MiniCollect Z serum separator tubes (Greiner Bio One, Monroe, NC, USA). 

### 4.8. Total Anti-ZIKV IgG Titer

Total anti-ZIKV IgG titer in serum was determined using sandwich ELISA as previously described [17]. Briefly, 96-well ELISA plates (Santa Cruz Biotechnology, Dallas, TX, USA) were coated overnight with whole ZIKV virus (100 ng/well in 50 µL/well) followed by 6 washes with PBS-T (1X PBS with 0.5% Tween 20). Wells were blocked with 50 µL/well blocking buffer (3% non-fat dry milk in PBS-T) for 1 h at room temperature. Serial dilution of serum was prepared in the blocking buffer and incubated (50 µL/well) in respective wells for 1 h at 37 °C. Similarly diluted pre-immune serum samples served as pre-immunization controls. Plates were washed 6 times with PBST followed by incubation with HRP conjugated Goat anti-mouse IgG (50 µL/well; 1:500 dilution) (Cell Signaling Technology, Danvers, MA, USA) for 1 h at 37 °C. Plates were washed six times with PBS-T and incubated with 50 µL/well TMB Blue 2-Component Peroxidase Substrate (SeraCare Life Sciences, Milford, MA, USA) for 10–15 min followed by stopping of reaction by equal volume of TMB Blue Stop Solution (SeraCare Life Sciences, Milford, MA, USA). Absorbance was measured at 600 nm and titers for each sample were determined as the dilution at which the absorbance was greater than the mean absorbance of saline injected control mice serum samples plus 3 times the standard deviation. 

### 4.9. ZIKV-neutralizing Antibody Titer 

ZIKV-neutralizing antibody titers were determined by standard plaque reduction neutralization test (PRNT). Serum samples were heat inactivated by incubating at 56 °C for 30 min followed by storage on ice. Two-fold serial dilutions were prepared starting at 10 by mixing the serum in dilution buffer (1XPBS supplemented with 0.1% FBS). Pre-immune serum samples served as pre-immunization controls. 100 pfu of ZIKV were mixed with each serum dilution in equal volumes, and binding of virus to the antibodies was allowed to occur for 1 h at 37 °C with intermittent mixing. Virus-antibody complex were plated on confluent Vero cells in 12-well plates and plates were incubated at 37 °C for 1 h with intermittent gentle shaking of the plate to ensure uniform distribution of the solution. Solution was removed from the culture plates and agarose overlay (EMEM with 1% low melting point agarose, 6% fetal bovine serum, and 1.2% penicillin/streptomycin) was plated on top of the cells. Plates were incubated for 4 day at 37 °C and 5% CO_2_ in a tissue culture incubator. Plaques were visualized by fixing and staining the cells with 0.1% crystal violet in 10% neutral buffered formalin for 4 h at room temperature. PRNT50 was determined as serum dilution which produced 50% reduction in the number of plaques compared to pre-immune serum. 

## Supplementary Materials

The following are available online at https://www.mdpi.com/2076-0817/8/4/188/s1, Figure S1: Infectivity of INA-inactivated ZIKV in Vero cells, Figure S2: Quantitative analysis of ZIKV infection in Vero cells. Figure S3: Infectivity of RNA isolated from INA-inactivated ZIKV in Vero cells.
