# Supplementary material for: Inactivation of Zika Virus by Photoactive Iodonaphthyl Azide Preserves Immunogenic Potential of the Virus
Source: Pathogens. 2019 Oct 12;8(4):188. doi: 10.3390/pathogens8040188 (PMC6963691; doi:10.3390/pathogens8040188)
Supplement: Supplementary file 1 [file pathogens-08-00188-s001.pdf]

**Supplementary Data**

Supplementary Figure S1

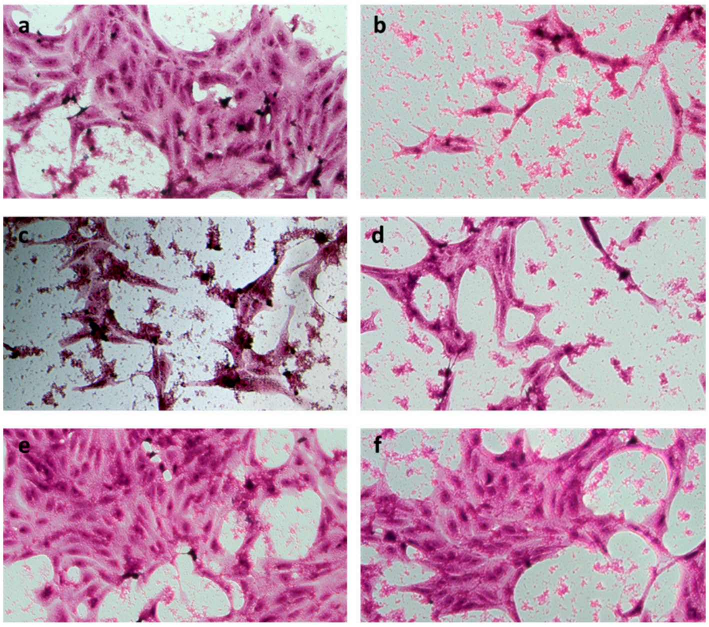


**Supplementary Figure S1**: **Infectivity of INA-inactivated ZIKV in Vero cells**: Crystal violet staining of Vero cells infected with various control and inactivated samples at passage 5 of serially passaged samples. (a) Uninfected; (b) ZIKV; (c) ZIKV + INA; (d) ZIKV + UV; (e) $INA_{50}$-iZIKV; (f) $INA_{100}$-iZIKV. Cells in uninfected (a), $INA_{50}$-iZIKV (e) and $INA_{100}$-iZIKV (f) grew to form monolayers, whereas cells were infected and majority died in ZIKV (b), ZIKV+INA (c) and ZIKV + UV (d) samples.

**Supplementary Figure S2**

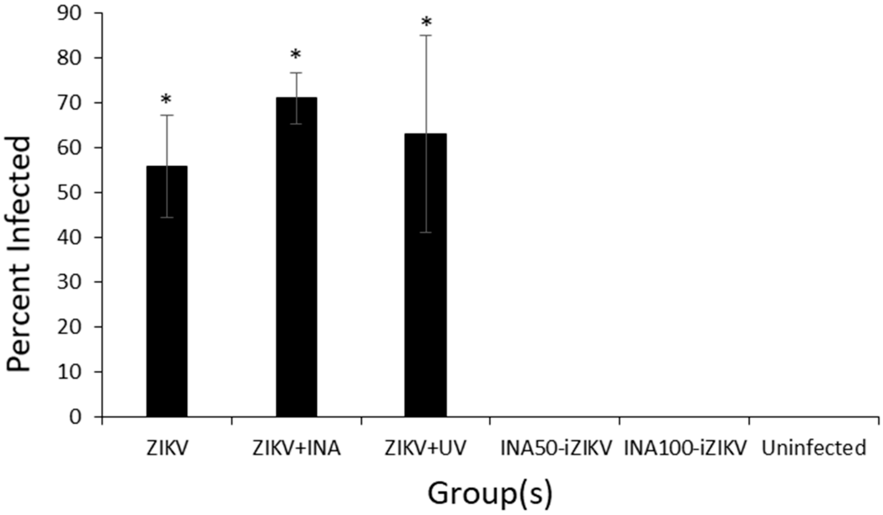


**Supplementary Figure S2**: **Quantitative analysis of ZIKV infection in Vero cells**: For each group, three representative microscopic fields at 20X objective were randomly selected and interpolated to ImageJ software. Total number of cells were counted using nuclear staining and number of infected cells were manually counted. Percent infection for each microscopic field was calculated and values are presented as Mean ± SD. No significant differences were observed between ZIKV, ZIKV+ INA (100μM) and ZIKV + UV, which were positive for ZIKV specific staining. No virus specific staining was observed in $INA_{50}$-iZIKV, $INA_{100}$-iZIKV and uninfected groups. All the three infection positive groups were statistically significant over that of the uninfected and INA100-ZIKV (* P value <0.01).

**Supplementary Figure S3**

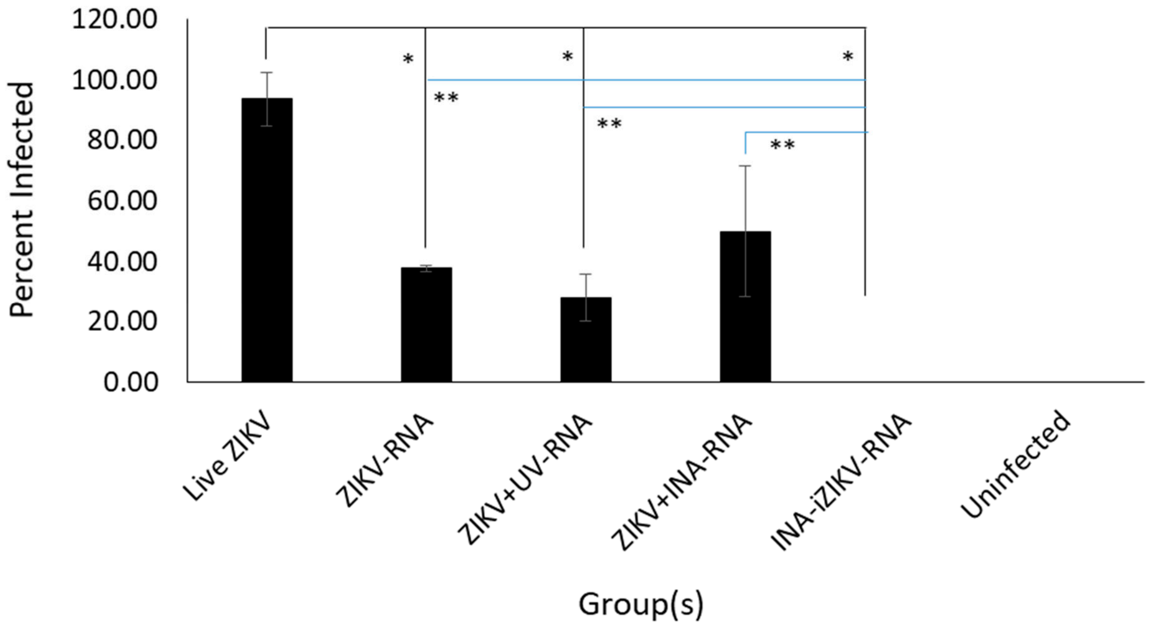


**Supplementary Figure S3**: **Infectivity of RNA isolated from INA-inactivated ZIKV in Vero cells**: For each group, representative microscopic fields at 40X objective were randomly selected and interpolated to ImageJ software. Total number of cells were counted using nuclear staining and number of infected cells were counted manually. Live ZIKV infected cells served as positive control for infection. Percent infection for each microscopic field was calculated and values are presented as Mean ± SD. No significant differences were observed between ZIKV-RNA, ZIKV + UV-RNA, and ZIKV+ INA (100μM)-RNA, which were positive for ZIKV specific staining. All these three groups were significantly (*) lower than the live ZIKV infection probably due to less number of cells infected from transfection as compared to most of the cells infected after infection with MOI=1 of live-ZIKV. All these three groups demonstrated significantly higher (**) infection over the INA-iZIKV-RNA groups which was negative for virus specific staining.
